# Decompensated liver cirrhosis as a rare consequence of long-term untreated panhypopituitarism after craniopharyngioma resection: a case report

**DOI:** 10.3389/fendo.2026.1758826

**Published:** 2026-05-18

**Authors:** Meijng Cui, Zhibin Yu, Qin Du, Zhongqiu Guo, Yanrong Chen, Min Liu, Ping Liu

**Affiliations:** 1 Department of Endocrinology and Metabolism, The Second Affiliated Hospital, CUHK-Shenzhen, Longgang District People’s Hospital of Shenzhen, Shenzhen, Guangdong, China; 2 Department of Orthopedics, The Second Affiliated Hospital, CUHK-Shenzhen, Longgang District People’s Hospital of Shenzhen, Shenzhen, Guangdong, China

**Keywords:** craniopharyngioma, growth hormone deficiency, hypopituitarism, liver cirrhosis, non-alcoholic fatty liver disease (NAFLD)

## Abstract

**Background:**

Panhypopituitarism is a common complication following craniopharyngioma surgery, and it is a known risk factor for metabolic disturbances, including non-alcoholic fatty liver disease (NAFLD). However, the progression from NAFLD to end-stage, decompensated liver cirrhosis in this context is exceptionally rare and seldom reported.

**Case presentation:**

We report the case of a 36-year-old male who presented with decompensated liver cirrhosis (ascites and portal hypertension) 13 years after resection of a craniopharyngioma. He had received no regular hormone replacement therapy for more than a decade. Notably, he had no postoperative hyperphagia, his body mass index was 21.1 kg/m2, and both fasting venous glucose (4.11 mmol/L) and glycated hemoglobin (4.7%) were normal. The workup for common etiologies of cirrhosis was negative, including viral hepatitis and the available autoimmune serologies. Endocrine evaluation confirmed severe panhypopituitarism, including central adrenal insufficiency, central hypothyroidism, central hypogonadism, and severe growth hormone deficiency (IGF-1 Z-score -2.7). Upper gastrointestinal endoscopy documented esophagogastric varices, and liver biopsy confirmed established cirrhosis with mild hepatocellular steatosis, supporting NAFLD as the most plausible underlying pathway. The patient was initiated on comprehensive hormone replacement (including hydrocortisone, levothyroxine, testosterone, and GH) and supportive care, with complete resolution of ascites at the 2-month follow-up.

**Conclusions:**

This case demonstrates that end-stage liver disease can be a severe, albeit rare, consequence of long-term, untreated panhypopituitarism. We conclude that severe GH deficiency, acting in synergy with other hormonal deficits, likely served as the critical driver for NAFLD progression to cirrhosis. This report underscores the necessity of comprehensive, lifelong endocrine management and metabolic monitoring for craniopharyngioma survivors to prevent irreversible organ damage.

## Introduction

Craniopharyngioma is a rare, benign intracranial tumor typically managed with surgical resection. However, due to its close anatomical relationship with the hypothalamic-pituitary axis, surgical intervention often results in irreversible pituitary damage, leading to hypopituitarism in 60%-90% of patients (1). This condition necessitates lifelong hormone replacement therapy.

In recent years, the metabolic sequelae of untreated hypopituitarism, including obesity, insulin resistance, and non-alcoholic fatty liver disease (NAFLD), have garnered significant clinical attention (2). NAFLD is the most common chronic liver disease globally, with a spectrum ranging from simple steatosis to non-alcoholic steatohepatitis (NASH), which can progress to fibrosis and cirrhosis (3). While the association between hypopituitarism and NAFLD is established, the progression to overt, decompensated liver cirrhosis secondary to long-term, unmanaged panhypopituitarism is exceptionally rare and seldom reported (4).

Among the multiple hormonal deficiencies, Growth Hormone (GH) deficiency is increasingly recognized as a critical factor in the pathogenesis of NAFLD and its progression to fibrosis. However, its role as a primary driver towards end-stage liver disease is often overlooked in clinical practice, partly due to its non-specific clinical presentation (5).

Herein, we report a rare case of a 36-year-old male who presented with decompensated liver cirrhosis 13 years after craniopharyngioma surgery. We trace the etiology to long-term, untreated panhypopituitarism—with a focus on the central role of GH deficiency—and review the associated pathophysiological mechanisms.

## Case presentation

### Patient information and chief complaint

A 36-year-old male presented to our digestive department with a 3-month history of abdominal distension, which had worsened and was accompanied by bilateral lower extremity edema. He reported no history of alcohol consumption, illicit drug use, or any family history of liver disease or other hereditary conditions. The patient denied any history of drug allergies.

### History of present illness

Three months prior to admission, the patient developed progressive abdominal distension. He was diagnosed with “liver cirrhosis and ascites” at a local hospital. The underlying etiology of the cirrhosis was not determined, and he was discharged after receiving symptomatic treatment (details unspecified). However, his symptoms of abdominal distension persisted, and he subsequently developed bilateral lower extremity edema and poor appetite.

Upon admission to our hospital, he reported significant fatigue, poor physical stamina, and social withdrawal, but denied nausea, vomiting, visual field defects, or symptoms of hyperthyroidism (e.g., palpitations, heat intolerance). He and his family specifically denied postoperative hyperphagia, and there was no history suggesting hypothalamic dysregulation of appetite or body temperature. On physical examination, no cushingoid features were observed, including moon facies or central obesity.

### Past medical and surgical history

The patient’s medical history was significant for delayed development. In 2004, at the age of 16, he presented with a lack of male secondary sexual characteristics and short stature (height below 160 cm). In 2011 (age 23), a cranial MRI at a local hospital revealed a 37×30 mm sellar mass, suggestive of a germ cell tumor.

He was subsequently transferred to a tertiary hospital in Beijing, where an enhanced pituitary MRI described an irregular, cystic-solid mass (approx. 4.0 cm × 3.2 cm) in the suprasellar region extending to the third ventricle, consistent with a craniopharyngioma. His pre-operative hormone status was unknown.

In July 2011, he underwent a craniotomy for tumor resection. The postoperative pathology confirmed the diagnosis of craniopharyngioma. Following surgery, he experienced a growth spurt of over 20 cm. Critically, the patient received no regular follow-up or hormone replacement therapy for over 13 years. He only began irregular, unmonitored oral prednisone (5 mg, three times daily) and levothyroxine (50 μg, once daily) six months prior to the current admission, prescribed at a local clinic for his fatigue.

### Physical examination

Upon admission, the patient was conscious but appeared fatigued with poor nutritional status. His height was 181 cm and weight 69 kg, corresponding to a body mass index of 21.1 kg/m2; waist circumference was not available from the preserved chart. His vital signs were stable. The skin was pale, and axillary and pubic hair were sparse. The cardiopulmonary examination was unremarkable. The abdomen was visibly distended, with positive shifting dullness confirming ascites. Bilateral pitting edema was present in the lower extremities. A genitourinary examination revealed bilateral testicular atrophy. No cushingoid features (e.g., moon facies or buffalo hump) were observed.

### Diagnostic assessment

#### Laboratory findings

Initial laboratory tests revealed pancytopenia (Hemoglobin 97 g/L, White Blood Cell count 3.6×10^9/L, Platelet count 36×10^9/L). Liver function tests were abnormal, showing mildly elevated Alanine Aminotransferase (ALT) 56 U/L, Aspartate Aminotransferase (AST) 48.6 U/L, total bilirubin 29.6 μmol/L, and borderline albumin 35 g/L. Coagulation studies indicated impaired hepatic synthesis, with a prolonged prothrombin time (PT) of 16.7 seconds (Activity 62%). Fasting venous glucose was 4.11 mmol/L and glycated hemoglobin was 4.7%, arguing against overt dysglycemia. Hypokalemia (3.30 mmol/L) was also noted and was considered most likely related to the patient’s pre-admission hydrochlorothiazide use (25 mg once daily).

Crucially, the lipid panel indicated significant mixed hyperlipidemia: Triglycerides 2.3 mmol/L (Ref: 0-1.7), Total Cholesterol 5.72 mmol/L (Ref: 0-5.2), HDL-Cholesterol 2.15 mmol/L (Ref: 1.16-1.42), and LDL-Cholesterol 3.53 mmol/L (Ref: 0-3.4).

Serum liver fibrosis markers were significantly elevated, including Hyaluronic Acid (260.31 ng/ml; Ref: 0-120.00) and Laminin (252.66 ng/ml; Ref: 0-130.00).

An extensive workup for common etiologies of liver disease was negative, including viral hepatitis (A, B, C, E), autoimmune hepatitis markers (ANA, AMA, and liver-kidney microsomal antibody [LKM]), EBV IgM, liver parasites, syphilis, and HIV antibodies. Smooth muscle antibody testing was not available in the preserved record. Other available autoimmune cholestatic markers, including gp210 and sp100 antibodies, were negative. Tumor markers (AFP, CEA) were within normal limits.

#### Endocrine function assessment

The endocrine evaluation confirmed panhypopituitarism (see **Table 1**).

**Table 1:** Baseline endocrine laboratory findings.

Parameter	Measured value	Reference range
Morning cortisol (8:00 AM) (Cortisol)	0.17	4.26–24.85 μg/dL
Adrenocorticotropic hormone (ACTH)	<1	7.2–63.4 pg/mL
Total thyroxine (T4)	73.00	66–181 nmol/L
Total triiodothyronine (T3)	0.53	1.3–3.1 nmol/L
Free thyroxine (FT4)	12.30	12–22 pmol/L
Free triiodothyronine (FT3)	2.15	3.1–6.8 pmol/L
Thyroid-stimulating hormone (TSH)	0.14	0.27–4.2 mIU/L
Prolactin (PRL)	3.64	3.81–22.7 ng/mL
Follicle-stimulating hormone (FSH)	<0.3	1.5–12.4 mIU/mL
Luteinizing hormone (LH)	<0.3	1.7–8.6 mIU/mL
Estradiol (E2)	<4.99	11.3–43.2 pg/mL
Progesterone (P)	0.08	<0.149 ng/mL
Testosterone (T)	<0.03	2.49–8.36 ng/mL
Growth hormone (GH)	0.33	0.05–2.40 ng/mL*
Insulin-like growth factor 1 Z-score (IGF-1 Z-score)	-2.7	-2.0 to 2.0

* The reference interval for random GH was provided by the local laboratory; due to pulsatile secretion, random GH values should not be used alone to diagnose adult GH deficiency.

- Adrenal Axis: Critically low morning cortisol at 0.17 μg/dl (Ref: 4.26-24.85) with suppressed ACTH <1 pg/ml (Ref: 7.2-63.4), confirming central adrenal insufficiency. (A 24-hour urine free cortisol test was 192.1 μg/24h; this was considered unreliable and falsely elevated due to interference from the patient’s recent prednisone intake).
- Thyroid Axis: Low Free T3 (2.15 pmol/L) and T3 (0.53 nmol/L) with an inappropriately low-normal TSH (0.14 mIU/L), indicative of central hypothyroidism.
- Gonadal Axis: Severely deficient sex hormones (Testosterone <0.03 ng/ml) with suppressed gonadotropins (FSH <0.3, LH <0.3 mIU/ml), confirming central hypogonadism.
- GH-IGF-1 Axis: Serum insulin-like growth factor 1 (IGF-1) was profoundly decreased (16.66 ng/mL), corresponding to an age- and sex-matched Z-score of -2.7 (reference: -2.0 to 2.0). In the context of a documented organic hypothalamic-pituitary disorder, multiple additional pituitary hormone deficiencies, and a markedly reduced IGF-1 Z-score, adult growth hormone deficiency was considered clinically established. Formal GH stimulation testing was deferred because the patient was acutely decompensated at presentation and was unlikely to tolerate provocative testing safely. Alternative provocative tests such as the glucagon stimulation test were also not pursued during the acute hospitalization for the same reason, and oral macimorelin was not used because it was not routinely available in our clinical setting.
- Posterior Pituitary: The patient reported polydipsia and polyuria (3–5 L/day), and his urine specific gravity was 1.006, consistent with central diabetes insipidus.

#### Imaging and other findings

An abdominal MRI (3.0T) confirmed established liver cirrhosis, splenomegaly, portal hypertension with collateral circulation, and significant ascites. Diffuse hepatic nodules were interpreted as regenerative nodules.

Upper gastrointestinal endoscopy revealed medium esophageal varices with red wale signs and GOV1 gastric varices, confirming clinically significant portal hypertension. Abdominal ultrasonography combined with liver elastography had also been performed during the hospitalization, but only the qualitative report rather than a standardized liver stiffness value was retained in the archived record.

**Figure 1:**
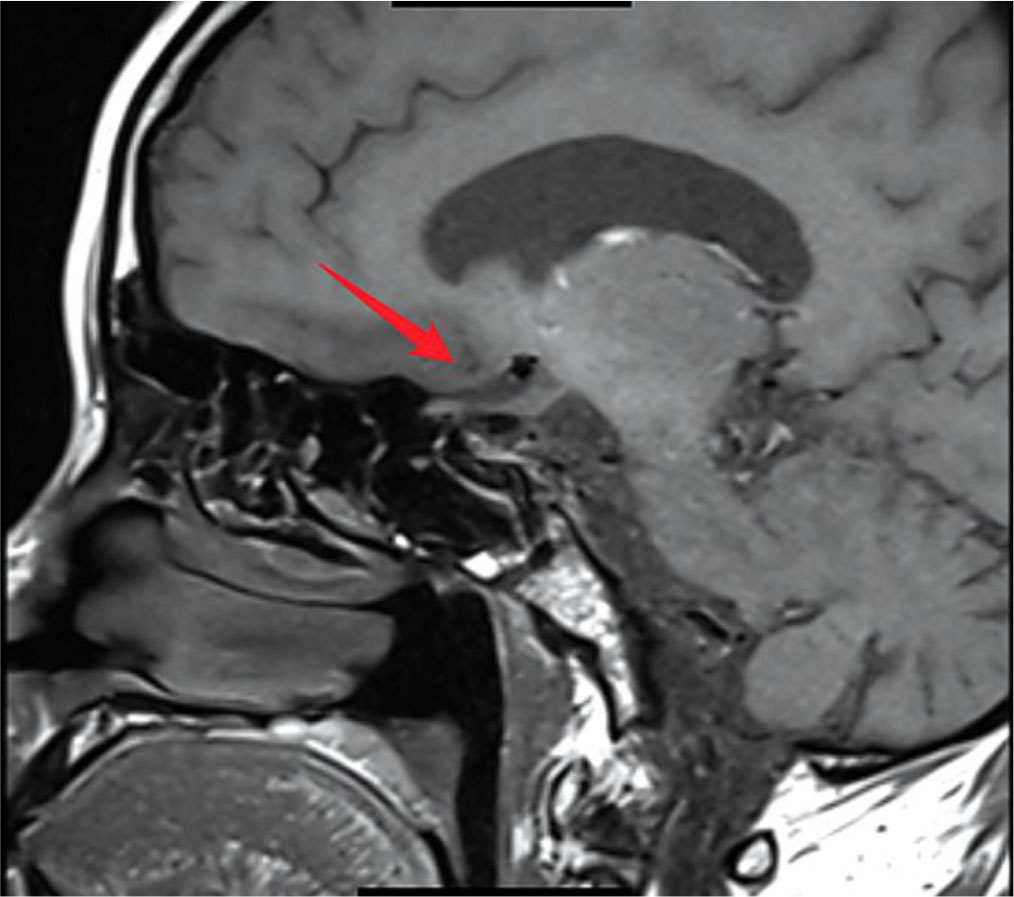
Post-operative pituitary MRI (sagittal view). A 3.0T contrast-enhanced T1-weighted sagittal image demonstrating significant post-operative changes in the sellar and suprasellar region. The arrow indicates abnormal soft tissue in the suprasellar area, consistent with post-surgical alterations following craniopharyngioma resection.

**Figure 2:**
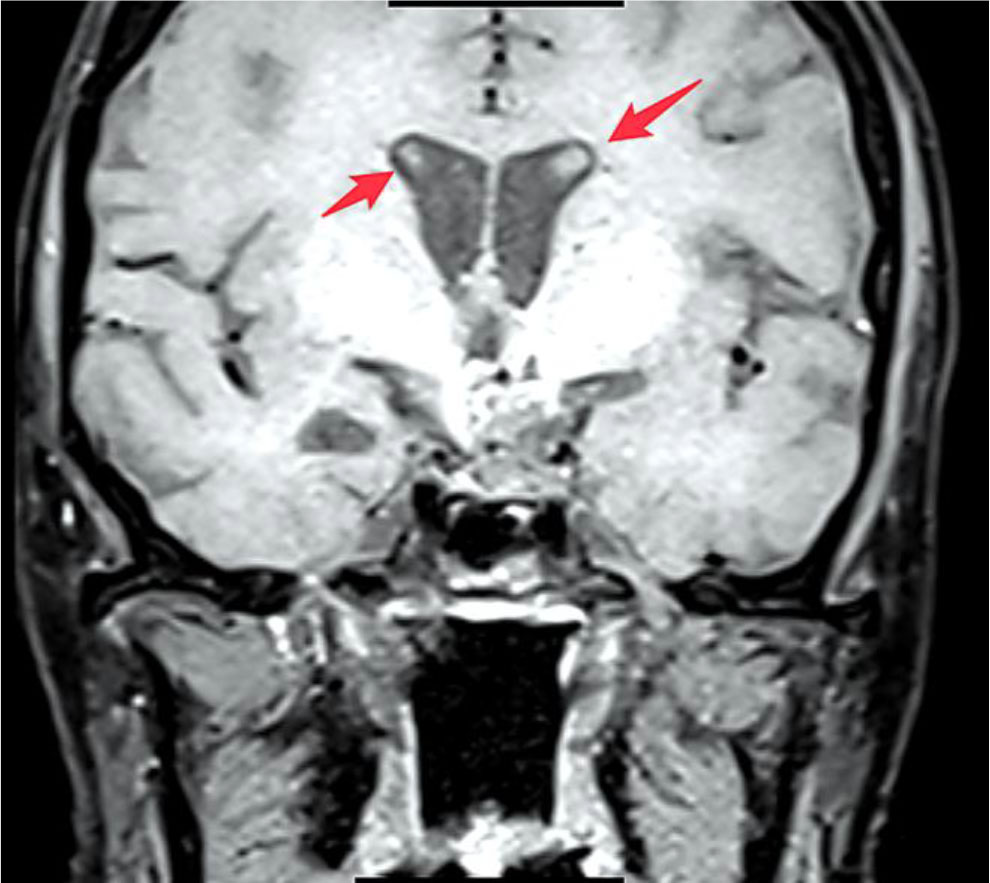
Post-operative pituitary MRI (coronal view). A 3.0T contrast-enhanced T1-weighted coronal image confirming post-surgical changes in the sellar/suprasellar region and demonstrating mild dilatation of the lateral ventricles (arrows).

A pituitary MRI (3.0T) revealed post-operative changes and a mixed-signal mass in the suprasellar region (**Figure 1**); tumor recurrence could not be definitively excluded. Mild dilation of the lateral ventricles was also noted (**Figure 2**).

Other findings included severe osteoporosis on a DXA scan (L1-L4 Z-score: -3.2) and delayed bone age (corresponding to a 15-year-old male).

#### Pathological findings

A review of the liver biopsy (performed at the local hospital) showed extensive disruption of the hepatic lobular architecture with diffuse pseudolobule formation. There was moderate-to-severe fibrous septa expansion with collagen deposition and a mild inflammatory infiltrate. Crucially, the microscopic examination also revealed mild hepatocellular steatosis (fatty change). The findings were consistent with established liver cirrhosis with features of portal hypertension.

### Final diagnosis

Based on the comprehensive assessment, the final diagnosis was panhypopituitarism after craniopharyngioma resection, manifested by secondary adrenal insufficiency, central hypothyroidism, central hypogonadism, severe adult growth hormone deficiency, central diabetes insipidus, and secondary osteoporosis.

He also had decompensated liver cirrhosis, most likely attributable to NAFLD arising from long-term untreated panhypopituitarism, complicated by ascites, splenomegaly, portal hypertension with esophagogastric varices, and hypokalemia likely related to prior hydrochlorothiazide exposure.

### Therapeutic intervention and follow-up

Upon transfer to our department, the patient was initiated on comprehensive hormone replacement and supportive care. This included hydrocortisone for adrenal insufficiency, levothyroxine sodium for hypothyroidism, desmopressin acetate for central diabetes insipidus, and testosterone undecanoate with injectable human chorionic gonadotropin (hCG) for hypogonadism. He also received calcium, active Vitamin D, and liver-protective/anti-fibrotic therapies. His condition improved, and he was discharged. Upon discharge, regular growth hormone (GH) replacement was also initiated.

At a 2-month follow-up visit, the patient’s ascites had completely resolved, and his general condition was markedly improved. His lipid profile also improved after hormone replacement, with triglycerides 0.64 mmol/L, total cholesterol 4.69 mmol/L, HDL-cholesterol 2.00 mmol/L, and LDL-cholesterol 2.39 mmol/L. No repeat liver biopsy was performed because of the invasive nature of the procedure and the patient’s early clinical improvement. Although follow-up endocrine laboratory data (e.g., IGF-1, free T3, and free T4) were not available, the temporal association between hormone replacement and clinical improvement suggests a contributory role of hypopituitarism in the patient’s decompensated liver disease.

## Discussion

We present a rare case of decompensated liver cirrhosis in a 36-year-old male, 13 years after surgical resection for craniopharyngioma. The etiology of his liver disease was initially obscure, presenting as cryptogenic cirrhosis. However, a comprehensive evaluation strongly indicates that the cirrhosis was the end-stage result of non-alcoholic fatty liver disease (NAFLD), driven by over a decade of severe, untreated panhypopituitarism.

The diagnostic challenge in this case was to connect the patient’s endocrine failure with his end-stage liver disease. The etiological diagnosis was established by: (1) systematically excluding common causes such as viral hepatitis, autoimmune hepatitis, and alcohol consumption (the patient denied any history of alcohol use); (2) identifying long-standing, profound panhypopituitarism, particularly severe Growth Hormone (GH) deficiency (IGF-1 Z-score -2.7); (3) the presence of significant hyperlipidemia (Triglycerides 2.3 mmol/L, Total Cholesterol 5.72 mmol/L); and (4) the definitive liver biopsy finding of established cirrhosis with concurrent mild hepatocellular steatosis (fatty change). This pathological finding provides a direct link, confirming that a fat-related liver injury process was the underlying driver of the cirrhosis.

The pathophysiology is likely a “multi-hit” process, with GH deficiency acting as the critical catalyst. First, GH and its effector, IGF-1, are essential for maintaining hepatic lipid homeostasis. GH deficiency disrupts this balance, promoting *de novo* lipogenesis and reducing fatty acid oxidation, leading to the accumulation of triglycerides in the liver (steatosis) (6). This mechanism is clinically supported in our patient by his overt hyperlipidemia and the steatosis found on pathology. Second, GH/IGF-1 deficiency accelerates the progression from simple steatosis to non-alcoholic steatohepatitis (NASH) and fibrosis. IGF-1 normally suppresses the activation of hepatic stellate cells (HSCs), the primary collagen-producing cells (7). The profound IGF-1 deficiency in this patient would have removed this inhibitory effect, leading to unchecked HSC activation and accelerated collagen deposition (fibrosis), as evidenced by his highly elevated liver fibrosis markers.

Furthermore, the detrimental effects of GH deficiency were amplified by the concurrent, severe deficiencies in other pituitary axes (8). The patient’s central hypothyroidism (low FT3/T3) is an independent risk factor for NAFLD, further impairing lipid metabolism and increasing oxidative stress. Similarly, his severe hypogonadism (Testosterone <0.03 ng/ml) is strongly associated with increased visceral adiposity and insulin resistance, contributing another “hit” to the liver. Together, these long-standing hormonal deficits likely created a sustained metabolic milieu favoring steatosis, fibrogenesis, and eventual cirrhosis.

This case presents several critical clinical implications. First, for patients presenting with “cryptogenic cirrhosis,” a detailed endocrine history, including any history of cranial surgery, radiation, or trauma, is essential. Panhypopituitarism should be considered in the differential diagnosis. Second, this case underscores the necessity of lifelong, comprehensive follow-up for craniopharyngioma survivors. This follow-up must not only monitor for tumor recurrence but also vigilantly manage all pituitary hormone deficiencies, including GH, which is often neglected in adult patients but is critical for metabolic health. The resolution of this patient’s ascites following comprehensive hormone replacement (including GH) highlights that correcting the underlying metabolic driver is paramount, even at the end stage.

Our observation is consistent with a recent Pituitary case report describing hypopituitarism-associated NAFLD in Sheehan syndrome that improved after growth hormone replacement therapy following a prolonged poor response to standard NAFLD care. Although the endocrine etiology differed, both cases support the concept that recognizing and correcting GH deficiency may be clinically relevant in selected hypopituitary patients with advanced metabolic liver disease.

We acknowledge several limitations of this case report. First, serial imaging studies and repeat liver biopsy were not available to longitudinally document the progression from simple steatosis to cirrhosis over the 13-year disease course or the histological response after hormone replacement. Nevertheless, the presence of residual hepatic steatosis in the cirrhotic biopsy is consistent with a metabolic etiology and supports this proposed pathway. In addition, follow-up endocrine laboratory measurements after hormone replacement therapy were unavailable, precluding a quantitative assessment of biochemical recovery. MRI proton spectroscopy and reproducible quantitative elastography data were also not preserved in the archived record, limiting more precise longitudinal quantification of steatosis and fibrosis.

In addition, although the patient had received supraphysiological doses of prednisone for approximately six months prior to presentation, which may have aggravated metabolic disturbances, this short-term exposure alone is unlikely to fully account for the development of end-stage cirrhosis. Taken together, these findings suggest that long-standing, untreated panhypopituitarism may have contributed to the progressive metabolic liver injury observed in this patient. However, a causal relationship cannot be definitively established based on a single case.

## Conclusions

This case demonstrates that decompensated liver cirrhosis can be a rare but severe long-term consequence of untreated panhypopituitarism following craniopharyngioma surgery. We conclude that severe, chronic Growth Hormone deficiency, acting in concert with other hormonal deficits (hypothyroidism, hypogonadism, and hypocortisolism), serves as a critical driver for the progression of NAFLD to end-stage cirrhosis. This case highlights the necessity for clinicians to consider endocrine dysfunction in the differential diagnosis of cryptogenic cirrhosis, particularly in patients with a history of cranial surgery or radiation. Finally, it underscores the importance of lifelong, comprehensive endocrine management for craniopharyngioma survivors, including GH replacement, to prevent irreversible metabolic complications such as end-stage liver disease.

## Data Availability

The original contributions presented in the study are included in the article/supplementary material. Further inquiries can be directed to the corresponding author.
